# Evaluation of a Real-Time PCR Test for the Detection and Discrimination of *Theileria* Species in the African Buffalo (*Syncerus caffer*)

**DOI:** 10.1371/journal.pone.0075827

**Published:** 2013-10-17

**Authors:** Mamohale E. Chaisi, Michiel E. Janssens, Lieve Vermeiren, Marinda C. Oosthuizen, Nicola E. Collins, Dirk Geysen

**Affiliations:** 1 Department of Veterinary Tropical Diseases, University of Pretoria, Pretoria, South Africa; 2 Department of Biology, National University of Lesotho, Roma, Lesotho; 3 Department of Biomedical Sciences, Institute of Tropical Medicine, Antwerp, Belgium; 4 Therapeutic Systems Central Europe, Terumo, Ikaroslaan, Zaventm, Belgium; Institut national de la santé et de la recherche médicale - Institut Cochin, France

## Abstract

A quantitative real-time PCR (qPCR) assay based on the *cox* III gene was evaluated for the simultaneous detection and discrimination of *Theileria* species in buffalo and cattle blood samples from South Africa and Mozambique using melting curve analysis. The results obtained were compared to those of the reverse line blot (RLB) hybridization assay for the simultaneous detection and differentiation of *Theileria* spp. in mixed infections, and to the 18S rRNA qPCR assay results for the specific detection of *Theileria parva*.

*Theileria parva*, *Theileria* sp. (buffalo), *Theileria taurotragi*, *Theileria buffeli* and *Theileria mutans* were detected by the *cox* III assay. *Theileria velifera* was not detected from any of the samples analysed. Seventeen percent of the samples had non-species specific melting peaks and 4.5% of the samples were negative or below the detection limit of the assay. The *cox* III assay identified more *T. parva* and *Theileria* sp. (buffalo) positive samples than the RLB assay, and also detected more *T. parva* infections than the 18S assay. However, only a small number of samples were positive for the benign *Theileria* spp. To our knowledge *T. taurotragi* has never been identified from the African buffalo, its identification in some samples by the qPCR assay was unexpected.

Because of these discrepancies in the results, *cox* III qPCR products were cloned and sequenced. Sequence analysis indicated extensive inter- and intra-species variations in the probe target regions of the *cox* III gene sequences of the benign *Theileria* spp. and therefore explains their low detection. The *cox* III assay is specific for the detection of *T. parva* infections in cattle and buffalo. Sequence data generated from this study can be used for the development of a more inclusive assay for detection and differentiation of all variants of the mildly pathogenic and benign *Theileria* spp. of buffalo and cattle.

## Introduction

*Theileria parva* is the causative agent of Corridor disease (theileriosis) in cattle in South Africa, and the African buffalo (*Syncerus caffer*) is the reservoir host. As theileriosis and other diseases carried by buffalo are a threat to farming communities in the endemic areas of the country [1], interaction between cattle and buffalo is limited in South Africa. Buffalo must test negative for *T. parva* before translocation, and this has resulted in an increased demand and cost of disease-free animals [2]. The tests used for the diagnosis of *T. parva* should therefore be sensitive and specific for accurate diagnosis.

*Theileria parva* usually co-occurs with other *Theileria* species in infected cattle and buffalo. These include the mildly pathogenic *T. mutans* and *T. buffeli*/*T. orientalis* which are usually carried asymtomatically, but under conditions of stress, malnutrition and immune-deficiency, can also cause disease, loss of production and may increase the severity of theileriosis in infected animals [3]. Some strains of *T. mutans* have been associated with severe disease in cattle [4] and invasion of the brain capillaries by this species can result in a form of benign bovine theileriosis known as turning sickness [5]. Other *Theileria* spp. of buffalo and cattle are *T. velifera* and *Theileria* sp. (buffalo). *Theileria velifera* was first described from cattle in 1964 [6] and is a mild pathogen of the African buffalo and cattle [3]. Very little is known about *Theileria* sp. (buffalo), it was first reported from a buffalo in Kenya [7]. It has not been reported in cattle and is therefore regarded as non-pathogenic and its vector is unknown. *Theileria taurotragi*, a parasite of eland (*Taurotragus oryx*), can also infect cattle. In South Africa *T. taurotragi* infection has been associated with bovine cerebral theileriosis and Tzaneen disease [8]. There are no reports of the occurrence of *T. taurotragi* from the African buffalo.

Polymerase chain reaction (PCR) assays are more sensitive and specific than microscopy and serological methods, and usually limit the subjectivity that occurs in the interpretation of results [9], [10]. Real-time PCR is easy to perform, less prone to contamination and reduces the time and labour required for attainment of results [11], [12]. A qPCR assay based on the 18S rRNA gene was recently developed [13] and is currently used, together with other diagnostic tests, for the diagnosis of *T. parva* infections in cattle and buffalo in South Africa. During the development of the qPCR, the 18S rRNA gene of *Theileria* sp. (buffalo) was sequenced and was found to be closely similar to that of *T. parva* [13]. While the hybridization probes developed by these authors can distinguish between *T. parva* and *Theileria* sp. (buffalo) amplicons, both species are amplified by the primers used in the qPCR assay [13], [14]. The sensitivity of the test in diagnosing *T. parva* from buffalo that are co-infected with *Theileria* sp. (buffalo) is therefore compromised. Alternative assays based on more informative molecular markers are needed to accurately detect and differentiate between pathogenic and non-pathogenic *Theileria* species in cattle and buffalo.

A nested qPCR assay based on the cytochrome oxidase subunit (*cox*) III gene has been developed for simultaneous detection and differentiation of six *Theileria* spp. in cattle samples by melting curve analysis using a single set of hybridization probes [15]. Both qPCR assays are based on fluorescence resonance energy transfer (FRET) technology which involves the use of sequence-specific oligonucleotide (hybridization) probes that are labelled with fluorescent dyes [16]. Amplification and hybridization occur in the same reaction. When both probes have hybridized to the PCR product, and are heated by slowly raising the temperature, the donor (anchor) probe absorbs light and transfers the resonance energy to the acceptor (sensor) probe. The temperature at which the hybridization probes are melted off the DNA strand (T_m_) can then be quantitatively measured in real-time by melting curve analysis [17] [18]. The sensor probe usually covers the variable target sequence, and therefore determines the T_m_. If the target DNA strand matches the sensor probe perfectly, the probe melts off at a higher temperature than when there is a mismatch and a single base mismatch between the sensor probe and mutant can reduce the T_m_ by up to 10°C [17].

The *cox* III qPCR assay [15] can detect and differentiate *T. parva*, *T. annulata*, *T. mutans*, *T. taurotragi*, *T. velifera* and *T. buffeli* in a single PCR reaction. The primers are genus-specific and amplify the *cox* III genes of all six *Theileria* spp. The probes hybridize to the PCR products of the different species but the sensor probe target region is variable for each species. Due to these nucleotide mismatches, the sensor probe will melt off the different amplicons at different temperatures which are species-specific. This allows for discrimination between the different species and mixed infections give clear species-specific peaks at different temperatures [15]. This assay was further modified to include the detection of *Theileria* sp. (buffalo) from buffalo samples. The aim of the current study was to evaluate the modified *cox* III qPCR assay for use in the detection and differentiation of *Theileria* spp. in mixed infections in buffalo. The results obtained were compared to those obtained by the reverse line blot (RLB) hybridization assay [19] which is also used to simultaneously detect and differentiate *Theileria* spp. in mixed infections, and to those of the 18S rRNA qPCR assay [13] for the specific detection of *T. parva*.

## Materials and Methods

### Ethics statement

This study was done in agreement with the South African National Parks (SANParks), represented by Dr Freek Venter (Specialist Head of Department: Conservation Services). The cattle samples from the Agricultural Research Council-Onderstepoort Veterinary Institute (ARC-OVI) were provided by Dr Fred Potgieter (former Senior Researcher, OVI). The protocol was approved by the Animal Use and Care Committee (AUCC) of the University of Pretoria (Number: V063-07).

### Samples and DNA extraction

A total of 224 blood samples (buffalo = 220: cattle = 4), collected either in EDTA tubes or on Whatman FTA® filter paper, were analysed. The buffalo samples originated from African buffalo in different game parks in South Africa and Mozambique (Table 1). The four cattle samples were obtained from the ARC-OVI. DNA was extracted using the QIAamp® DNA Extraction Kit (QIAGEN, Southern Cross Biotechnologies) for blood on filter paper, or the High Pure PCR Template Preparation kit (Roche Diagnostics, Mannheim, Germany) for whole blood samples in EDTA according to the manufacturers' protocols, and stored at −20°C until further analysis. Both methods have previously been used in our laboratory to isolate DNA from blood samples effectively.

**Table 1:** Origin and no. of samples analysed by the modified *cox* III qPCR assay.

Place of origin	Country (Province)	No. of samples
Kruger National Park (KNP)	SA* (Mpumalanga and Limpopo)	54
Hluhluwe-iMfolozi Game Park (HIP)	SA (KwaZulu-Natal)	100
Agricultural Research Council - Onderstepoort Veterinary Institute (ARC-OVI)	SA (Gauteng)	4**
Greater Limpopo Transfrontier Game Park (GLTP)	Mozambique	33
Others:	SA	
Addo Elephant Game Park (AEGP)	(Eastern Cape)	13
Marakele National Park	(Limpopo)	5
Ithala National Park	(KwaZulu-Natal)	8
Vaalbos National Park	(Northern Cape)	6
Kwanare Game Park	(Mpumalanga)	1
**TOTAL**		**224**

* SA – South Africa.

** Cattle.

### Polymerase Chain Reaction

A nested PCR protocol was used for the amplification of a fragment of the *cox* III gene of the parasite. Forward primer F3Cox (5′-AAGATGAATCCGATTTGATGA-3′) and reverse primer MJCoxF3 (5′-AAATGGACTATGTAAGTTAACCTAT-3′) were used in a primary conventional hot start PCR reaction using the BIOMETRA (Whatman Biometra, Gottingeng, Germany) thermocycler. The reaction mixture contained 1 µl yellow sub (GENEO BioProductions, Hamburg, Germany), 5 µl of 1× Go Taq buffer (Promega), 1.65 mM MgCl_2_, 2.5 mM dNTPs, 0.4 µM of each primer, 0.5 U *Taq* Polymerase (Promega), 5 µl (∼100 ng) DNA and PCR grade water to a total volume of 25 µl. The cycling conditions included an initial hold at 84°C for 10 s, initial denaturation at 92°C for 4 min, amplification of 40 cycles each of denaturation at 92°C for 30 s, annealing at 56°C for 45 s and extension at 72°C for 60 s, and a final extension at 72°C for 10 min. Each PCR run consisted of 32 field samples, together with a positive control (*Theileria parva* positive sample) and a negative control (water). Each sample was tested once. All primary PCR products were analysed by a nested *cox* III qPCR assay protocol using the Rotor Gene 3000 machine (Corbett Research, Australia). Each nested PCR reaction contained 0.5 µl of the primary PCR product, 5 µl of 1× Go Taq buffer (Promega), 1.65 mM MgCl_2_, 2.5 mM dNTPs, 0.66 µM of primer FCox (5′-CAACATTGTTAAAGCTATCCAA-3′) and 0.13 µM of primer nRCox (5′-TTATAGTACAGGATTAGATAC-3′), together with 0.5 µM each of the modified Anchor probe Cox1-6FAM (5′-ATTGGatgacattaTAtTtctatattttaaCaGGAc-3′) and Sensor probe Cox1-Cy5 (5′-AttcaTtacacGTatgtgCtggaag-3′), 5 U *Taq* polymerase and water to a total volume of 25 µl. Capital letters in the anchor and sensor probe sequences represent locked nucleic acids (LNAs). The programme included a hold at 95°C for 15 min, 40 cycles each of denaturation at 95°C for 30 s, annealing at 56°C for 45 s, extension at 72°C for 60 s. Melting curves were generated by heating the samples from 33°C to 99°C with a heating rate of 1°C/min. Fluorescence was measured at 640 nm.

Plasmid DNA from *cox* III clones (obtained from the Department of Biomedical Sciences, Institute of Tropical Medicine, Antwerp, Belgium) of the following species were used as positive controls: *Theileria* sp. (buffalo) clone 1.5 originated from a buffalo isolate from South Africa; *T. parva* Katete clone 1.5 was obtained from a bovine isolate (used as a vaccine strain) from the Eastern province of Zambia; *T. taurotragi* N355 clone 2.7 was obtained from a bovine isolate from the Eastern province of Zambia; *T. buffeli* M2138 clone 538 was from an imported bovine in Butare, Rwanda; *T. velifera* C914 clone 2.8 and *T. mutans* C914 clone 2.2 were from a mixed infection sample obtained from a bovine in the Eastern province of Zambia. A total of 36 samples were included in each run, consisting of primary PCR products from 27 field samples, 6 plasmid positive control samples, a negative control containing molecular grade water, and the positive and negative controls from the nested PCR. Each sample was analysed once and selected samples were further characterized by cloning and sequencing.

### Cloning and sequencing

Based on the qPCR results, 17 samples (Table 2) were selected for further characterization. Primary PCR products were purified using the High Pure PCR product purification kit (Roche Diagnostics, Mannheim, Germany) according to the manufacturer's instructions. The samples originating from the Addo Elephant Game Park (AEGP) (n = 7) had single *T. buffeli* infections and their *cox* III genes were amplified and PCR products were directly sequenced. The PCR product from the sample from buffalo KNP/102 [13] was directly sequenced; it was also cloned and sequenced. PCR products from the remaining nine samples were cloned and sequenced. Ligations and transformations were done using the pCR2.1 TOPO cloning vector (Invitrogen, Carlsbad, USA) as recommended by the manufacturer. Ninety-three recombinants obtained from these samples were screened using the *cox* III qPCR assay as described above. Recombinant clones containing *cox* III inserts were sequenced using primers F3Cox, MJCoxF3 and nRcox. Plasmid extraction and sequencing were done at the Genetic Service Facility, University of Antwerp, Belgium and at Inqaba Biotechnologies, South Africa.

**Table 2:** Results of the RLB and modified *cox* III qPCR assays for selected samples and clones.

Sample	RLB result	Clone	*cox*III qPCR result (T_m_°C)	Phylogenetic classification
**HIP A2**	***Theileria*** ** sp. (buffalo),**		***Theileria sp.*** **(buffalo), ** ***T. parva***	
	***T. buffeli, T. mutans***	1.1	*NSP (*T. velifera/T. parva*) (47.2)	*T. velifera*
		1.7	*NSP (*T. taurotragi*/*T.mutans*) (62)	*T. velifera*-like
		1.8	*T. taurotragi* (56°C)	*T. mutans-*like A
		1.9	*Theileria* sp. (buffalo) (40.5)	*Theileria* sp. (buffalo)
**KNP K8**	***T. velifera, T. mutans***		***T. parva, T. taurotragi*** (55.3 )	
		2.2	*NSP *(T. taurotragi/T. mutans)* (61.8)	*T. velifera*-like
**KNP K4**	***T. mutans***		***T. parva, T. taurotragi*** (55.8)	
		3.4	*T. taurotragi* (55.5°C)	*T. mutans-like* A
		3.8	* NSP (*T. taurotragi/T. mutans*) (59.3)	*T. mutans*-like B
		3.9	* NSP (*T. taurotragi/T. mutans)* (60.7 )	*T. mutans*-like C
**OVI 8227**	***T. mutans***		***T. parva, T. mutans***	
		4.7	* NSP (*Theileria* sp. (buffalo)/*T. velifera*) (42.8)	** Inconclusive
		4.10	* NSP (*T. taurotragi/T. mutans*) (59)	*T. mutans*-like B
**KNP E20**	***T. velifera, T. mutans***		***T. parva,*** * ***NSP (T. taurotragi/T. mutans*** **) (56.5)**	
		5.10	*T. taurotragi* (56 )	*T. mutans*-like A
**KNP 102**	***T. parva***		***T. parva***	*T. parva*
		6.1	*T. parva* (48.3 )	*T. parva*
		6.2	*T. parva* (48 )	*T. parva*
**KNP E10**	***T. mutans***		* **NSP (** ***Theileria*** ** sp. (buffalo)/** ***T. velifera*** **) (42.3), ** ***T. taurotragi*** ** (55.5)**	
		7.1	* NSP (*T. taurotragi*/*T. mutans*) (62 )	*T. mutans*-like C
**KNP E2**	***T. velifera, T. mutans***		* ***NSP (Theileria*** ** sp. (buffalo)/** ***T. velifera*** **)(42), ** ***T. taurotragi***	
		8.5	* NSP *(T. taurotragi/T. mutans*) (61.2 )	*T. velifera*-like
		8.6	*T. taurotragi* (55.3 )	*T. mutans*-like A
**KNP C8**	***T. velifera, T. mutans***		* **NSP ** ***(T. taurotragi/T. mutans)*** ** (62 ), ** ***T. parva*** **, ** ***T. mutans***	
		9.1	*T. mutans* (64 )	*T. mutans*
		9.4	* NSP (*T. taurotragi/T. mutans*) (62 )	*T. mutans-like C*
**KNP B22**	***T. parva, T.mutans, T.velifera***		***T. parva, Theileria sp. (buffalo)***	
		10.10	*T. mutans* (63.5 )	*T. mutans*-like C
**Addo 65**	***T. buffeli***		*T. buffeli* (53.2)	*T. buffeli*-like
**Addo 66**	***T. buffeli***		*T. buffeli* (53.3)	*T. buffeli*-like
**Addo 69**	***T. buffeli***		*T. buffeli* (53.1)	*T. buffeli*-like
**Addo 70**	***T. buffeli***		*T. buffeli* (53.7)	*T. buffeli*-like
**Addo 73**	***T. buffeli***		*T. buffeli* (53.5)	*T. buffeli*-like
**Addo 74**	***T. buffeli***		*T. buffeli* (53.5 )	*T. buffeli*-like
**Addo 76**	***T. buffeli***		*T. buffeli* (53.2)	*T. buffeli*-like

* Indicates a non-species specific melting peak located between the peaks for the species indicated in brackets.

** Did not cluster with any of the other sequences and its identity could not be established.

### Sequence and phylogenetic analyses

The *cox* III sequences were assembled and edited using GAP4 of the Staden software package (version 1.6.0 for Windows) [20] and representative sequences have been deposited in GenBank under accession numbers KF512672–KF512681. A BLASTn homology search of GenBank was done using the consensus sequences. MAFFT version 5 [21] was used to align the new sequences with *cox* III gene sequences of the control clones, and with published *Theileria cox* III gene sequences from GenBank (*T. parva* Z23263, *T. parva* AB499089, *T. orientalis* AB499090, *T. annulata* U32225). The alignment was manually edited using BioEdit (version 7) [22]. The genetic distances between the sequences were estimated by determining the number of base differences between sequences using MEGA4 [23]. Phylogenetic trees were constructed using MEGA4 for neighbor-joining analysis with 1000 bootstrap replicates [24]; PAUP* (v4b10) [25] for maximum-parsimony and maximum likelihood methods, and MrBayes v.3.1.2 [26] for Bayesian inference, accessed via the Computational Biology Service Unit, Cornell University (http://mafft.cbsuapps.tc.cornell.edu/mrbayes.aspx). The TrN+I+G substitution method was determined as the best fit model by Modeltest v.3.7 [27] and used in the likelihood and Bayesian analyses. The trees were rooted using the *cox* III gene sequence of *Theileria annulata* (U32225) and consensus trees were edited using MEGA 4.

### Comparison of the cox III qPCR assay with the RLB assay and the 18S rRNA qPCR assay

The samples (n = 224) were analysed by the RLB hybridization assay for the simultaneous detection and differentiation of *Theileria* spp as described [19]. The *Theileria* and *Babesia* species- and genus-specific probes used were as previously described [28]. Additionally, 206 samples were analysed for the specific detection of *T. parva* using the 18S rRNA qPCR assay as previously described [13]. The occurrence of *T. parva* in these samples was compared to that of the *cox* III qPCR and RLB hybridization assays.

## Results

### cox III qPCR assay

**Figure 1:**
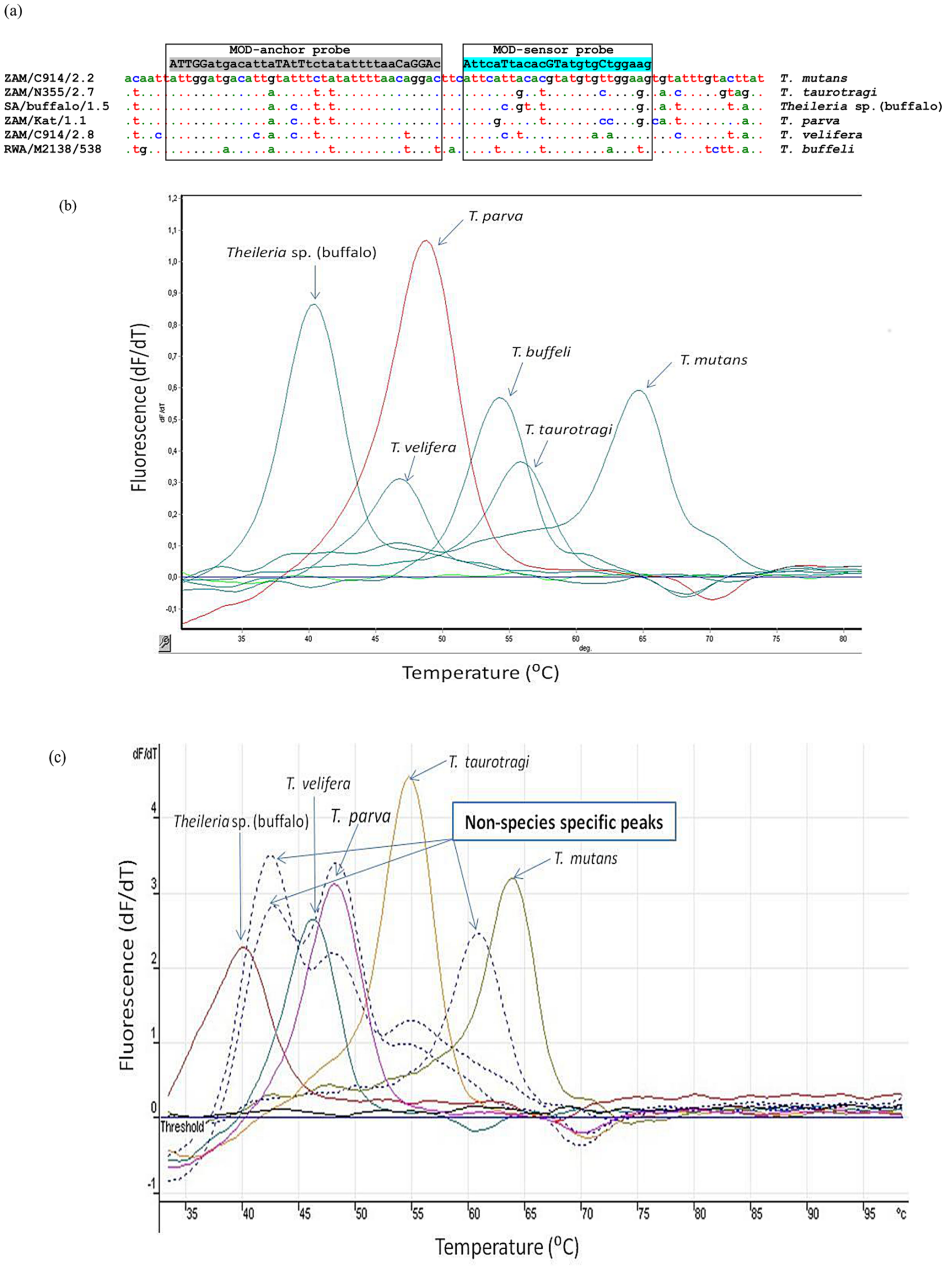
Sequence alignment and melting curve analysis of the plasmid controls of the *cox* III gene. (a) Sequence alignment showing the number of mismatches in the modified FRET anchor and sensor probe areas in the target area of the *cox* III gene of the different *Theileria* species. (b) Melting curve analysis of the *cox* III gene plasmid controls of *Theileria* spp. as determined by the *cox* III qPCR assay. Melting peaks shown are for *Theileria* sp. (buffalo) (39.7±0.5°C), *T. velifera* (46.0±0.4°C), *T. parva* (48.4±0.3°C), *T. buffeli* (53.7±0.1°C), *T. taurotragi* (54.7±0.8°C) and *T. mutans* (63.9±0.4°C). (c) Non-species specific peaks (arrows) were observed from some samples. No fluorescence was detected from the negative (water) control.

Amplicons of approximately 980 bp were obtained from primary PCR amplification of the *cox* III gene of *Theileria* spp. The hybridization probes used in the *cox* III real-time PCR assay allow for the detection and discrimination of the different species based on differences in their melting temperatures (T_m_). The sequences of the modified anchor and sensor probes and *Theileria* spp. controls are indicated in Figure 1(a). Melting peaks obtained from the secondary amplicons (approximately 680 bp) were analysed by comparing them with those of the control clones as illustrated in Figure 1(b). As the melting peaks can shift slightly from run to run, the mean and standard deviation of the T_m_ of the control plasmids and analysed samples were determined.

**Figure 2:**
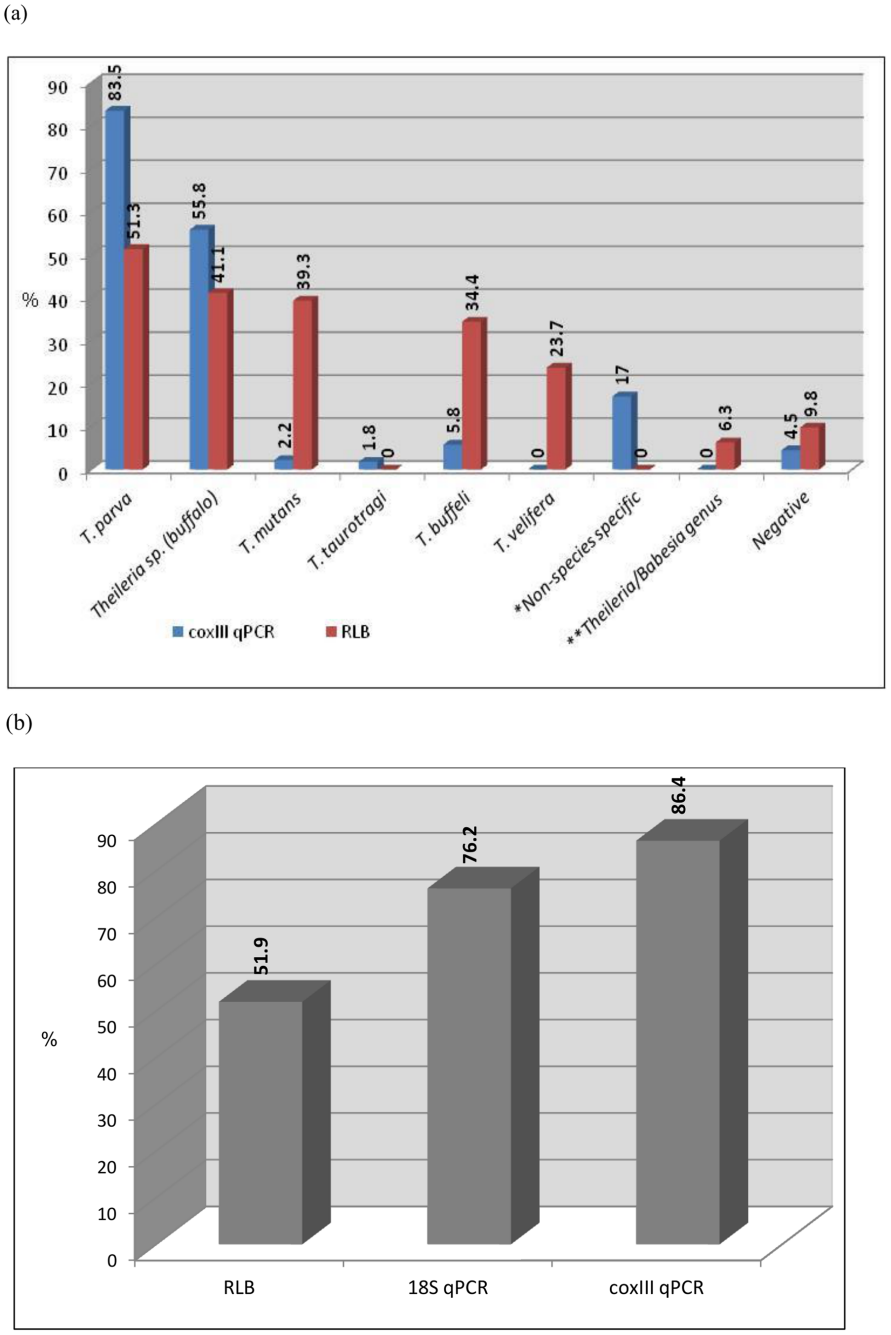
Occurrence of *Theileria* species infections in buffalo and cattle samples from South Africa and Mozambique. (a) As determined by the RLB and *cox* III qPCR assays (n = 224). The number of samples with non-species specific melting temperatures on the *cox* III qPCR assay (*) and those that hybridized only with the *Theileria*/*Babesia* genus-specific probes using the RLB assay (**) are shown. (b) Comparison of the RLB, 18S qPCR and *cox* III qPCR assays for detection of *T. parva* (n = 206).

*Theileria parva* and *Theileria* sp. (buffalo) were the most commonly detected species in the field samples from buffalo and cattle, with prevalences of 83.5% and 55.8%, respectively (Figure 2a). *Theileria taurotragi*, *T. buffeli* and *T. mutans* were identified in 1.8%, 5.8% and 2.2% of samples, respectively (Figure 2a). *Theileria velifera* was not identified in any of the samples, and 4.5% of the samples were negative or below the detection limit of the assay. Additionally, 17% of the samples had non-species specific peaks which were located between the peaks of *Theileria* sp. (buffalo) and *T. velifera*, *T. velifera* and *T. parva*, or *T. taurotragi* and *T. mutans* (Figure 1c).

### Comparison of the cox III qPCR assay with the 18S qPCR and RLB hybridization assays

The results of the *cox* III qPCR assay were compared to those obtained by the RLB hybridization assay for the simultaneous detection and differentiation of *Theileria* spp. in buffalo and cattle (n = 224). *Theileria parva* (51.3%) and *Theileria* sp. (buffalo) (41.1%) were also identified as the most commonly occurring species by the RLB assay (Figure 2a). However, the RLB assay detected more infections of *T. mutans* (39.3%) and *T. buffeli* (34.4%) than the *cox* III qPCR assay, 23.7% of samples were positive for *T. velifera* and no *T. taurotragi* infections were detected by the RLB assay. Of the 14 samples that only had a *Theileria/Babesia* genus-specific signal on the RLB assay, 11 showed mixed *Theileria* spp. infections and 3 were negative on the *cox* III qPCR assay (Figure 2a).

The *cox* III qPCR assay results were also compared with those of the RLB and 18S qPCR assays for the specific detection of *T. parva*. This species was detected in 107 (51.9%), 157 (79.2%) and 178 (86.4%) of the 206 samples analysed by the RLB, 18S qPCR and *cox* III qPCR assays, respectively (Figure 2b). All samples that tested positive for *T. parva* by the RLB assay were also positive when analysed with the two qPCR assays. *Theileria parva* was identified from buffalo in all localities by the three assays, mainly as mixed infections, except for the AEGP where only *T. buffeli* was identified.

### Sequencing and phylogenetic analyses

**Figure 3:**
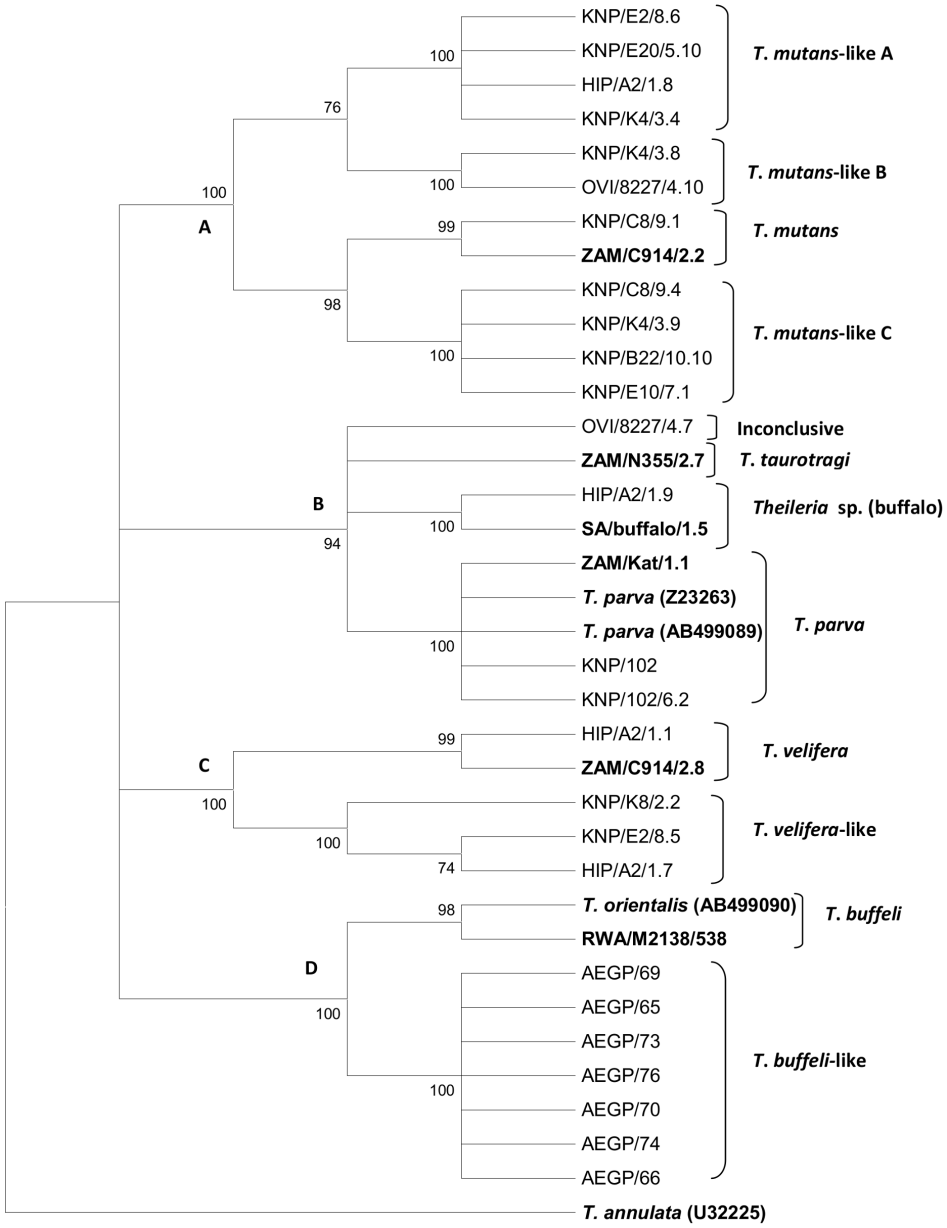
Phylogenetic relationships of the *cox* III gene sequence variants of *Theileria* spp. The figure shows sequences identified in this study (black) with *Theileria* control sequences (bold) and published *Theileria* spp. (italics). Bootstrap values indicate the degree of support for each cluster. The tree was outgroup rooted using the *cox* III gene sequence of *T. annulata*.

Samples selected for further characterization (Table 2) included those in which the *cox* III qPCR assay: (i) indicated the presence of non-species specific melting peaks; (ii) failed to detect *T. mutans* and *T. velifera* infections as identified by the RLB assay and/or (iii) detected *T. taurotragi* infections. Selected *T. buffeli* positive samples from AEGP were also sequenced as there was a slight shift in the T_m_ of these samples from that of the *T. buffeli* control clone (RWA/M2138/538) from cattle. The *cox* III gene from the *T. parva* positive control sample (KNP/102) was also sequenced in order to determine any variations within the *cox* III gene of *T. parva*. The *cox* III gene PCR products of these samples were cloned and the clones were subjected to the *cox* III qPCR assay. The following species were detected from 93 clones analysed by the *cox* III qPCR assay: *T. parva* (13); *T. mutans* (10); *T. buffeli* (7); *Theileria* sp. (buffalo) (9); *T. velifera* (2) and *T. taurotragi* (17). In addition, 14 clones had non-species specific melting peaks which were either between the peaks of *Theileria* sp. (buffalo) and *T. velifera*, *T. velifera* and *T. parva*, *T. mutans* and *T. taurotragi* and 21 clones were negative or below detection limit. Based on these results, selected clones were sequenced and a total of 26 new *cox* III gene sequences were obtained. A BLASTn homology search revealed that the generated sequences had closest homology with *cox* III gene sequences of *T. parva* Z23263 (81–100%), *T. parva* AB499089 (79–100%) and *T. orientalis* AB499090 (78–88%). Phylogenetic trees were constructed from a total of 36 *cox* III gene sequences (26 new sequences obtained in this study, 6 control sequences and 4 sequences obtained from GenBank). The groupings in the trees generated by the different algorithms were similar and were supported by high bootstrap values and posterior probabilities (for Bayesian analysis). A representative phylogenetic tree constructed using the NJ method is shown in Figure 3. The sequences grouped into four distinct clades, with 12 *cox* III sequence variants (Figure 3).

**Figure 4:**
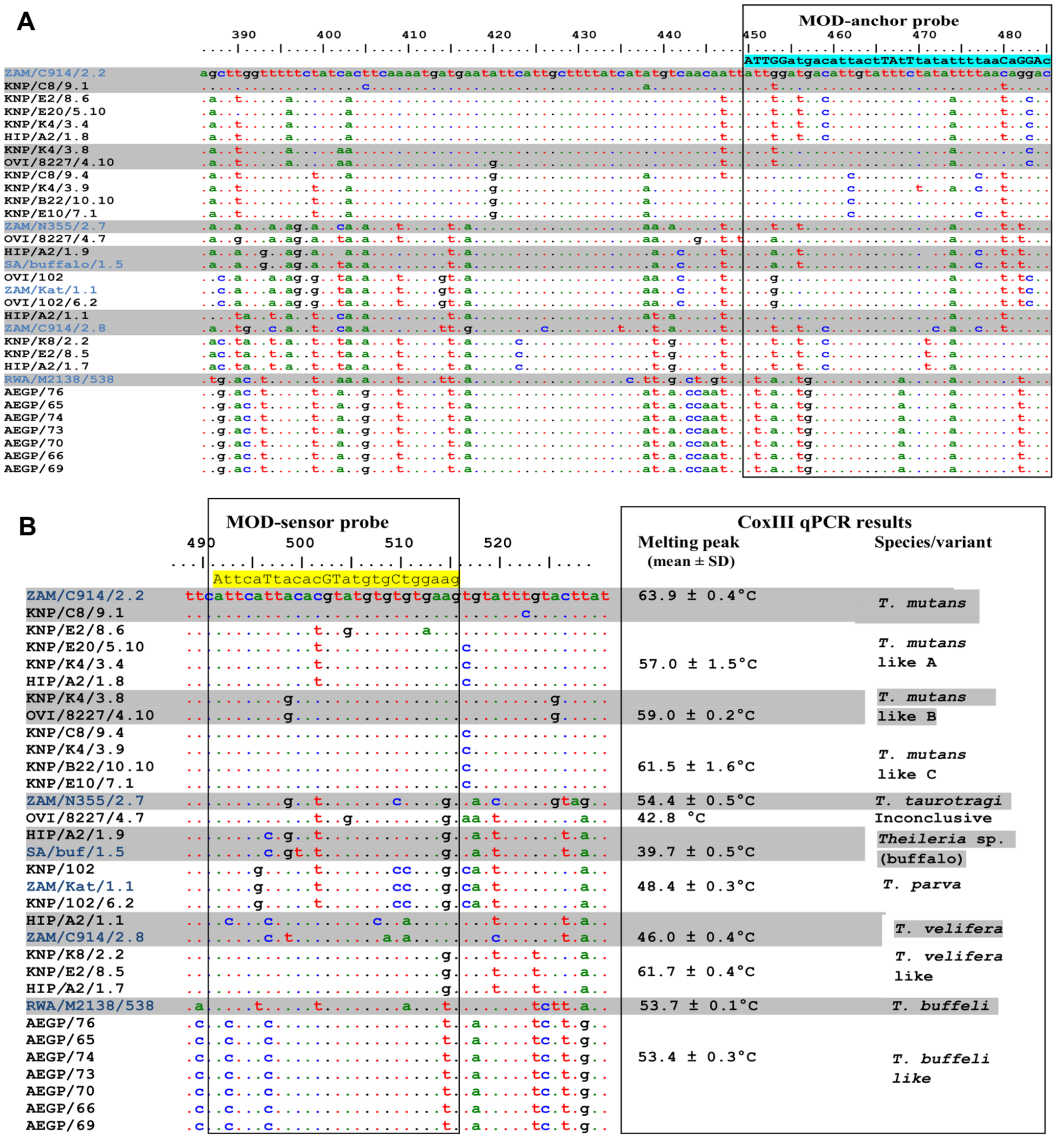
Alignment of the probe area of the *cox* III gene sequences. The modified anchor (light blue shading) and sensor (yellow shading) probe sequences are indicated. *cox* III sequences were obtained from clones from control samples (blue text) and clones from selected African buffalo samples. Differences are based on the *cox* III sequence of *T. mutans* (ZAM/C9142.2). The identified *cox* III sequence variants are indicated.

The *T. mutans* group (clade A) contained four sequence variants and was the most polymorphic group. Sequence KNP/C8/9.1 from a buffalo sample, was identical to the *T. mutans* control *cox* III sequence ZAM/914/2.2 from a bovine sample. Clone KNP/C8/9.1 tested positive for *T. mutans* by the *cox* III qPCR assay. The original sample, KNP/C8, tested positive for *T. mutans* and *T. velifera* when analysed by the RLB hybridization assay (Table 2), but no *T. velifera cox* III sequences were identified by the qPCR assay from this sample. The three *T. mutans*-like *cox* III variants were designated *T. mutans*-like A–C (Figure 3). All of the clones from which these sequences were obtained had non-species specific melting temperatures that were between those of *T. taurotragi* and *T. mutans* when analysed by the *cox* III qPCR assay (Table 2). The four *T. mutans*-like A sequences were all derived from buffalo samples. The two *T. mutans*-like B sequences from clones KNP/K4/3.8 and OVI/8227/4.10 were derived from a buffalo sample and a bovine sample respectively, and the four *T. mutans*-like C sequences were buffalo-derived. Nucleotide differences of 1–3 bp in the modified sensor probe between *T. mutans* and *T. mutans*-like *cox* III variants resulted in a shift in T_m_ of up to 8°C (Figure 4), and as a result, clones with *T. mutans*-like *cox* III sequences yielded non-species specific melting peaks when analysed by the *cox* III qPCR assay.

Clade B consisted of *cox* III gene sequences of the pathogenic *Theileria* spp. (*T. taurotragi* and *T. parva*) as well as those of *Theileria* sp. (buffalo). None of the new sequences grouped with the *T. taurotragi cox* III control sequence (ZAM/N355/2.7) obtained from a bovine sample from Zambia. Although sequence OVI/8227/4.7, obtained from a bovine at the OVI, grouped in Clade B, it did not cluster with any of the other sequences and its identity could not be established; it was therefore indicated as inconclusive. The PCR product generated from this clone had a melting peak between *Theileria* sp. (buffalo) and *T. velifera* when tested using the *cox* III qPCR assay, and the field sample was positive for *T. mutans* by the RLB assay. A similar melting peak profile to that from OVI/8227/4.7 was obtained from sample KNP/E2 (Table 2), but a clone with a similar profile was not obtained and therefore the identity of samples with such profiles could not be confirmed. The sequence from clone HIP/A2/1.9 was identical to the *cox* III sequence of the *Theileria* sp. (buffalo) control clone (SA/buffalo/1.5). The *T. parva cox* III sequences (KNP/102 and KNP/102/6.2) from buffalo 102 were identical to the *T. parva* (Muguga stock) *cox* III sequences from cattle, Z23263 [29] and AB499089 [30], and to the *cox* III sequence (ZAM/Kat/1.1) of the *T. parva* control used in this study (Figure 3). Samples KNP/102 and ZAM/Kat tested positive for *T. parva* by the *cox* III PCR and *T. parva* was identified in the original field samples using the RLB assay.

Clade C contained the *T. velifera cox* III sequences from cattle (ZAM/C914/2.8) and buffalo (HIP/A2/1.1), and *T. velifera*-like *cox* III sequences (KNP/K8/2.2, KNP/E2/8.5, HIP/A2/1.7) which were all derived from buffalo. *T. velifera* was only identified (using the RLB assay) in two of the three field samples from which these clones were obtained, KNP/K8, KNP/E2, while sample HIP/A2 had a mixed infection of *T. mutans*, *T. buffeli* and *Theileria* sp. (buffalo) (Table 2). The failure of the RLB assay to detect *T. velifera* in sample HIP/A2 might be due to low parasitemia. Although the *cox* III qPCR assay failed to detect *T. velifera* in the field samples, the sequence of clone HIP/A2/1.1 grouped together with that of the *T. velifera* control sequence. The melting peak of this clone was between those of *T. parva* and *T. velifera* (Table 2). The nucleotide difference in the probe area of the two sequences (Figure 4) is likely to have resulted in the shift of the melting peak. Although sequences KNP/K8/2.2, KNP/E2/8.5, HIP/A2/1.7 (designated as *T. velifera*-like) grouped together with the *T. velifera* sequences (Figure 3), their melting peaks were between those of *T. taurotragi* and *T. mutans* with T_m_ similar to those of the *T. mutans*-like C sequences (Table 2).

The last clade (D) was the *T. buffeli* group. The *cox* III sequences of the seven samples from the AEGP were identical and differed from the published *cox* III gene sequence of *T. orientalis* (AB499090) [30] and the sequence of the *T. buffeli* control clone (RWA/M2138/538). The AEGP sequences were therefore designated as *T. buffeli*-like *cox* III sequences. Although there are three bp differences in the sensor probe areas of RWA/M2138/538 and the AEGP sequences, their melting temperature was almost identical (0.3°C difference). All the field samples from which these sequences were derived tested positive for *T. buffeli* using the RLB assay.

Figure 4 indicates that the presence of diverse *cox* III species variants in *Theileria* species of buffalo can affect the diagnostic results of the *cox* III qPCR assay as the melting peaks of these novel variants do not correspond with the melting peaks of known species (Table 2). The melting temperature results (Table 2) indicated that samples and clones that had non-species specific melting peaks positioned between the *T. taurotragi* and *T. mutans* peaks contained *T. mutans*-like B and C or *T. velifera*-like variants. There was a range of melting peaks for *T. mutans*-like C melting profiles, from 60.7–63.5°C; the highest of these overlapped with the *T. mutans* melting peak profile. The melting peaks of *T. mutans*-like C clones also overlapped with those of *T. velifera*-like clones, and therefore it would be impossible to distinguish these variants using melting temperature analysis. Sequences identified as *T. taurotragi* by the cox III qPCR were the *T. mutans*-like A variant. Samples and clones with a non-species specific peak positioned between *T. velifera* and *T. parva* peaks were probably *T. velifera* as identified in clone HIP/A2/1.1 (Table 2).

## Discussion and Conclusion

FRET technology has previously been used for the development of diagnostic assays to simultaneously identify co-infecting piroplasmids (*Theileria* and *Babesia* spp.) in different hosts. One such qPCR assay [31] is based on the 18S rRNA gene and can simultaneously detect and differentiate *Babesia bovis*, *Babesia divergens*, *Babesis major*, *Babesia bigemina*, *Theileria annae* and an unidentified *Theileria* sp. infection in bovines. Another previously reported qPCR assay [32], also based on the 18S rRNA gene, detects and differentiates *Babesia gibsoni*, *Babesia canis canis*, *Babesia canis vogeli* and *Babesia canis rossi* in canines. In addition to the identification of *T. parva* in infected animals, the 18S qPCR assay used in South Africa [13] can simultaneously detect *T. taurotragi* and *T. annulata* when the *Theileria*-genus primer set is used. A *cox* III qPCR assay was previously developed for the simultaneous detection and differentiation of *T. parva* and five co-infecting *Theileria* spp. (namely *T. annulata*, *T. velifera*, *T. taurotragi*, *T. mutans* and *T. buffeli*) in infected cattle based on differences in their melting temperatures [15]. This assay was later modified, by the development of new primer and probe sets, to include the detection of *Theileria* sp. (buffalo) in buffalo (unpublished results). In this study the modified *cox* III qPCR assay was compared with the RLB assay for the ‘universal’ detection and discrimination of *Theileria* spp. in cattle and buffalo samples, and with the 18S qPCR assay for the specific detection of *T. parva*.

The analytical sensitivities of the qPCR assays for the detection of *T. parva* were determined as 100% at parasitemia of 8.79×10^−4^% for the 18S qPCR assay [13], and between 4.1×10^−5^ and 4.1×10^−4^% for the *cox* III qPCR assay [15], indicating that the latter assay might be slightly more sensitive than the former. This is supported by our study as more *T. parva* infections were identified by the *cox* III qPCR than the 18S rRNA qPCR assay. Both qPCR assays have previously been reported as being more sensitive than the 18S- and *cox* III RFLP-PCR assays in detecting *T. parva* [13], [15].

The *cox* III qPCR assay was also more sensitive than the RLB assay in the detection of *T. parva* and *Theileria* sp. (buffalo) infections. All the samples that had tested positive for *T. parva* by the RLB assay were also positive when using the two qPCR assays. Additionally, the qPCR assays detected *T. parva* in some samples that were negative for this species by the RLB assay. Similarly, the same samples were positive for *T. parva* by the 18S rRNA assay and the *cox* III assay, although the *cox* III identified more *T. parva* positive samples than the 18S assay. Failure of the RLB assay to detect *T. parva* infections that were detected by the qPCR assays is likely due to low parasitemia as real-time PCR has previously been reported to be more sensitive than the RLB assay in detecting *Theileria* and *Babesia* spp. [13], [33].

We previously reported the occurrence of *T. parva* and other *Theileria* spp. in buffalo from different localities in South Africa and Mozambique using the RLB assay [28]. The current results confirmed the presence of these species in buffalo originating from different game parks in South Africa, with *T. buffeli* being the only *Theileria* species infecting buffalo in the AEGP. Our results also indicate that the *cox* III gene of *T. parva* is highly conserved, although more sequences from field samples need to be analysed in order to confirm this result. Any variation in the probe sequence target area may compromise accurate identification of this pathogen as any nucleotide differences are likely to cause a shift in the melting peak. The occurrence of 18S rRNA sequence variants in the V4 hypervariable region of *T. parva* in the South African cattle and buffalo population have been identified [14], and some of these variants gave melting peak profiles that shifted from that of the standard *T. parva* control sample [14].

The *cox* III qPCR assay is less laborious, less time-consuming and more cost-effective than the RLB assay as it requires the use of a single hybridization probe pair to detect all the *Theileria* spp., whereas the RLB requires a probe for each species or genotype that is identified. It therefore has the potential of replacing or complementing the RLB assay for the simultaneous detection of *Theileria* spp. of cattle in the future. However, the *cox* III qPCR assay used in this study was shown to be less specific than the RLB in the detection of other *Theileria* spp. that infect the African buffalo. Sequence and phylogenetic analyses of the *cox* III gene in our study indicated the presence of a single *T. parva* genotype in cattle and buffalo, and therefore the *cox* III qPCR assay can specifically detect *T. parva* infections in these hosts where the parasitemia is above the detection limit of the assay. However, sequence polymorphism in the *cox* III genes of the other *Theileria* species decreases the specificity of the assay for these species; hence their apparent low prevalences as indicated by the *cox* III qPCR results, and the discrepancies between *cox* III qPCR and RLB assay results. The sequences reported here were generated by PCR amplification and sequencing of cloned amplicons, and it is therefore possible that *Taq* polymerase errors may have contributed to the micro-heterogeneity observed in the *cox* III gene sequences, However, it is likely that most of the sequence diversity is real since the nucleotide differences are too frequent and not random enough across blocks of phylogenetically related sequences to be explained by *Taq* polymerase errors. The sequence data generated from this study can therefore be used to design a more species and/or variant specific assay.

The identification of *T. taurotragi* by the *cox* III qPCR assay in buffalo was unexpected. To our knowledge this species has never been isolated from the African buffalo. In addition, the RLB assay did not detect *T. taurotragi* in any of these samples. The RLB results were confirmed by sequence analysis: none of the *cox* III sequences derived from buffalo samples grouped together with the *T. taurotragi* control sequence. Reanalysis of the original qPCR results indicated that: organisms previously identified as *T. taurotragi* would be identified as the *T. mutans*-like A variant; the samples that had non-species specific melting peaks positioned between the *T. taurotragi* and *T. mutans* peaks contained *T. mutans*-like B and C or *T. velifera*-like variants; and the samples that had non-species specific melting peaks positioned between *T. velifera* and *T. parva* are likely to be positive for *T. velifera*. The shift observed in the melting peaks is due to nucleotide variations in the probe area of the different *T. mutans*-like and *T. velifera*-like sequences found in buffalo. These unique melting peak profiles were obtained from different samples and therefore provide evidence of the existence of these species variants in buffalo. The identity of the non-species specific peaks observed between those of *Theileria* sp. (buffalo) and *T. velifera* could not be established as only one sequence was obtained (OVI/8227/4.7) which could not be assigned to a specific group by phylogenetic analysis. Further studies are needed to determine if sequence OVI/8227/4.7 represents a unique sequence in cattle.

As with the 18S rRNA gene [34], the greatest heterogeneity in the *cox* III gene was observed within the *T. mutans* group. Three *T. mutans*-like sequence variants were obtained for both 18S and *cox* III genes, however, a direct comparison between the two genes could not be made as different samples were analysed in the two studies and pure parasite stocks are not available. Unlike with the 18S rRNA gene where all the variants were exclusively obtained from buffalo, we identified a *T. mutans*-like B *cox* III variant (OVI/8227/4.10) from a bovine. The identification of *T. velifera* in cattle and buffalo, and a *T. velifera*-like variant in buffalo is concurrent with our previous results on the 18S rRNA gene [34]. The identity of the species and/or variant with melting temperatures between those of *Theileria* sp. buffalo and *T. velifera* could not be established. We can only speculate that these are probably *T. velifera* or *T. velifera*-like organisms as *Theileria* sp. (buffalo) was specifically identified by the *cox* III qPCR assay. Although the *cox* III qPCR assay can accurately distinguish between the different *Theileria* spp. in cattle samples [15], failure of the assay to accurately distinguish between the different species variants present in buffalo is a limitation of the test. Our results further indicate the importance of the identification of all local sequence variants of a gene before the development of a diagnostic assay. The design of species-specific primers and probes has been restricted by the lack of *cox* III sequence data from the public sequence databases. Sequence data obtained from this study will therefore allow for the design of new primers and probes for effective differentiation between the different species and their variants.

In conclusion, the modified *cox* III qPCR is sensitive and specific for the detection of *T. parva* infections in cattle and buffalo, and the sensitivity and specificity of the assay for the identification of benign and non-pathogenic *Theileria* spp. in the African buffalo could be improved by the development of primers from a conserved area of the gene and probes from variable areas of the gene. However, there is extensive sequence variation within the *cox* III gene of *Theileria* spp. of the African buffalo. Although the gene is a good marker in phylogenetic studies of closely related species, it might not be a suitable gene for use in a diagnostic assay, particularly in *Theileria* spp. of buffalo where there is a lot of variation. It is possible that analysis of more samples could reveal more variation as the *cox* III gene is a fast evolving gene. As more sequence data are obtained it may prove increasingly difficult to distinguish unambiguously between closely related parasite species within buffalo using a single set of hybridization probes.

## References

[1] Uilenberg G (1999) Immunization against diseases caused by *Theileria parva*: A review. Trop Med Int Health 4: A12–20. 10540307 10.1046/j.1365-3156.1999.00446.x

[2] Collins NE, Allsopp MTEP, Allsopp BA (2002) Molecular diagnosis of theileriosis and heartwater in bovines in Africa. Trans R Soc Trop Med Hyg 96: S217–S224. 12055842 10.1016/s0035-9203(02)90079-9

[3] Norval RAI, Perry BD, Young AS (Eds.) (1992) The Epidemiology of Theileriosis in Africa. Academic Press, London, UK, 41 pp.

[4] Saidu MN (1981) *Theileria mutans* in Nigeria: clinical records of prevalence and experimental infection in calves. In: Ivin, A.D., Cullingham, M.P., Young, A.S. (Eds.), Advances in the control of Theileriosis. The Hague: Martinus Nijoff, pp. 86–87.

[5] Seifert HSH (1996) Benign bovine theileriosis. In: Tropical Animal Health. Kluwer Academic Publishers, The Netherlands. pp. 193.

[6] Uilenberg G, Schreuder BE, Mpangala C (1964) *Haematoxeus veliferus*, n. g., n. sp., parasite incertae sedis du sang de bovins a Madagascar. Revue d'Elevage et de Medecin Veterinaire des Pays Tropicaux 17: 655–662.

[7] Allsopp BA, Baylis HA, Allsopp MT, Cavalier-Smith T, Bishop RP, et al (1993) Discrimination between six species of *Theileria* using oligonucleotide probes which detect small subunit ribosomal RNA sequences. Parasitology 107: 157–165. 8414670 10.1017/s0031182000067263

[8] De Vos AJ, Roos JA (1984) The isolation of *Theileria taurotragi* in South Africa. The Ondestepoort Journal of Veterinary Research 8: 149–153. 6801567

[9] Figueroa JV, Buening GM (1995) Nucleic acid probes as a diagnostic method for tick-borne hemoparasites of veterinary importance. Vet Parasitol 57: 75–92. 7597795 10.1016/0304-4017(94)03112-a

[10] Zarlenga DS, Higgins J (2001) PCR as a diagnostic and quantitative technique in veterinary parasitology. Vet Parasitol 101: 215–230. 11707298 10.1016/s0304-4017(01)00568-4

[11] Bell A, Ranford-Cartwright L (2002) Real-time quantitative PCR in parasitology. Trends Parasitol 18: 338. 12377276

[12] Jaton K, Bille J, Greub G (2006) A novel real-time PCR to detect *Chlamydia trachomatis* in first-void urine or genital swabs. J Med Microbiol 55: 1667–1674. 17108270 10.1099/jmm.0.46675-0

[13] Sibeko KP, Oosthuizen MC, Collins NE, Geysen D, Rambritch NE, et al (2008) Development and evaluation of a real-time polymerase chain reaction test for the detection of *Theileria parva* infections in Cape buffalo (*Syncerus caffer*) and cattle. Vet Parasitol 155: 37–48. 18514421 10.1016/j.vetpar.2008.03.033

[14] Mans BJ, Pienaar R, Latif AA, Potgieter FT (2011) Diversity in the 18S SSU rRNA V4 hyper-variable region of *Theileria* spp. in Cape buffalo (*Syncerus caffer*) and cattle from southern Africa. Parasitology 1–14. 10.1017/S0031182011000187 21349232

[15] Janssens ME (2009) Molecular biological tools for the immunization and diagnosis of *Theileria parva*. PhD Thesis. Faculteit Wetenschappen. Universiteit Anwerpen. Belgium.

[16] Reuter M, Kupper Y, Schmitz A, Breuer JP, Wend U, et al (2005) Detection of new single nucleotide polymorphisms by means of real time PCR. J Genet 84: 341–345. 16385169 10.1007/BF02715807

[17] Caplin BE, Rasmussen RP, Bernard PS, Wittwer CT (1999) The most direct way to monitor PCR amplification for quantification and mutation detection. Biochemica 1: 5–8.

[18] Op de Buijsch RAM, de Vries JE, Loots WJC, Landt O, Wijnen PAHM, et al (2005) Genotyping of the PXR A11156C polymorphism with locked nucleic acid containing fluorogenic probes. TPJ 5: 72–74. 10.1038/sj.tpj.6500299 15772695

[19] Gubbels JM, Vos AP, Weide M, Viseras J, Schouls LM, et al (1999) Simultaneous detection of bovine *Theileria* and *Babesia* species by reverse line blot hybridization. J Clin Microbiol 37: 1782–1789. 10325324 10.1128/jcm.37.6.1782-1789.1999 PMC84950

[20] Staden R, Beal KF, Bonfield JK (2000) The staden package, 1998. Methods Mol Biol 132: 115–130. 10547834 10.1385/1-59259-192-2:115

[21] Katoh K, Kuma K, Toh H, Miyata T (2005) MAFFT version 5: Improvement in accuracy of multiple sequence alignment. Nucl Acids Res 33: 511–518. 15661851 10.1093/nar/gki198 PMC548345

[22] Hall TA (1999) BioEdit: a user-friendly biological sequence alignment editor and analysis program for Windows 95/98/NT. Nucl Acid Symp Ser 41: 95–98.

[23] Tamura K, Dudley J, Nei M, Kumar S (2007) MEGA4: Molecular evolutionary genetics analysis (MEGA) software version 4.0. Mol Biol Evol 24: 1596–1599. 17488738 10.1093/molbev/msm092

[24] Felsenstein J (1985) Confidence limits on phylogenies: An approach using the bootstrap. Evolution 39: 783–791. 28561359 10.1111/j.1558-5646.1985.tb00420.x

[25] Swofford DL (2003) PAUP*. Phylogenetic Analysis Using Parsimony (*and other methods). Version 4b10. Sinauer Associates, Sunderland, Massachusetts.

[26] Ronquist F, Huelsenbeck JP (2003) MrBayes 3: Bayesian phylogenetic inference under mixed models. Bioinformatics 19: 1572–1574. 12912839 10.1093/bioinformatics/btg180

[27] Posada D, Crandall KA (1998) MODELTEST: testing and model of DNA substitution. Bioinformatics 14: 817–818. 9918953 10.1093/bioinformatics/14.9.817

[28] Chaisi ME, Sibeko KP, Collins NE, Potgieter FT, Oosthuizen MC (2011) Identification of *Theileria parva* and *Theileria* sp. (buffalo) 18S rRNA gene sequence variants in the African buffalo (*Syncerus caffer*) in southern Africa. Vet Parasitol 182: 150–162. 21700394 10.1016/j.vetpar.2011.05.041

[29] Kairo A, Fairlamb AH, Gobright E, Nene V (1994) A 7.1 kb linear DNA molecule of *Theileria parva* has scrambled rDNA sequences and open reading frames for mitochondrially encoded proteins. EMBO J 13: 898–905. 8112303 10.1002/j.1460-2075.1994.tb06333.x PMC394889

[30] Hikosaka K, Watanabe Y, Tsuji N, Kita K, Kishine H, et al (2010) Divergence of the mitochondrial genome structure in the apicomplexan parasites, *Babesia* and *Theileria* . Mol Biol Evol 27: 1107–1116. 20034997 10.1093/molbev/msp320

[31] Criado-Fornelio A, Buling A, Pingret JL, Etievant M, Boucraut-Baralon C, et al (2009) Hemoprotozoa of domestic animals in France: Prevalence and molecular characterization. Vet Parasitol 159: 73–76. 19013719 10.1016/j.vetpar.2008.10.012

[32] Wang C, Ahluwalia SK, Li Y, Gao D, Poudel A, et al (2010) Frequency and therapy monitoring of canine babesia spp. infection by high-resolution melting curve quantitative FRET-PCR. Vet Parasitol 168: 11–18. 19931290 10.1016/j.vetpar.2009.10.015

[33] Bhoora R, Franssen L, Oosthuizen MC, Guthrie AJ, Zweygarth E, et al (2009) Sequence heterogeneity in the 18S rRNA gene within *Theileria equi* and *Babesia caballi* from horses in South Africa. Vet Parasitol 159: 112–120. 19019541 10.1016/j.vetpar.2008.10.004

[34] Chaisi ME, Collins NE, Potgieter FT, Oosthuizen MC (2013) Sequence variation identified in the 18S rRNA gene of *Theileria mutans* and *Theileria velifera* from the african buffalo (*Syncerus caffer*). Vet Parasitol 191: 132–137. 22940566 10.1016/j.vetpar.2012.08.005

